# Engineering enzyme activity using an expanded amino acid alphabet

**DOI:** 10.1093/protein/gzac013

**Published:** 2022-11-12

**Authors:** Zachary Birch-Price, Christopher J Taylor, Mary Ortmayer, Anthony P Green

**Affiliations:** School of Chemistry, Manchester Institute of Biotechnology, University of Manchester, Manchester, M1 7DN, UK; School of Chemistry, Manchester Institute of Biotechnology, University of Manchester, Manchester, M1 7DN, UK; School of Chemistry, Manchester Institute of Biotechnology, University of Manchester, Manchester, M1 7DN, UK; School of Chemistry, Manchester Institute of Biotechnology, University of Manchester, Manchester, M1 7DN, UK

**Keywords:** non-canonical amino acids, genetic code expansion, directed evolution

## Abstract

Enzyme design and engineering strategies are typically constrained by the limited size of nature’s genetic alphabet, comprised of only 20 canonical amino acids. In recent years, site-selective incorporation of non-canonical amino acids (ncAAs) via an expanded genetic code has emerged as a powerful means of inserting new functional components into proteins, with hundreds of structurally diverse ncAAs now available. Here, we highlight how the emergence of an expanded repertoire of amino acids has opened new avenues in enzyme design and engineering. ncAAs have been used to probe complex biological mechanisms, augment enzyme function and, most ambitiously, embed new catalytic mechanisms into protein active sites that would be challenging to access within the constraints of nature’s genetic code. We predict that the studies reviewed in this article, along with further advances in genetic code expansion technology, will establish ncAA incorporation as an increasingly important tool for biocatalysis in the coming years.

## Introduction

Enzymes are powerful biological catalysts that can display exceptional efficiencies and selectivities for a wide variety of reactions. As enzymes can be produced from sustainable feedstocks and are biodegradable, their use as industrial biocatalysts is increasingly viewed as an attractive alternative to traditional chemical processes. In addition, the high reaction selectivities achievable with enzymes often alleviate the requirement for costly protection and deprotection steps or removal of side products and can enrich the chiral purity of products (Turner, 2009; Bornscheuer *et al.*, 2012). Significantly, the catalytic properties of enzymes can be tailored through directed evolution to generate biocatalysts with an expanded substrate range, improved activities and selectivities or enhanced stability under viable process conditions. To engineer biocatalysts for non-natural reactions, the mechanistic promiscuity of enzymes can also be enhanced through protein engineering (Coelho *et al.*, 2013; Biegasiewicz *et al.*, 2018). Alternatively, the rapidly developing field of computational *de novo* enzyme design holds great promise for accessing novel non-biological catalytic activities, which can be enhanced via laboratory evolution to generate highly active and selective catalysts (Crawshaw *et al.*, 2021; Lovelock *et al.*, 2022). However, the mechanistic diversity of these designed enzymes is restricted by the small selection of functional groups in nature’s alphabet of canonical amino acids. One method of overcoming this challenge is to incorporate non-canonical amino acids (ncAAs) bearing reactive side chains into enzyme active sites, unlocking previously inaccessible areas of chemical space (Agostini *et al.*, 2017; Won *et al.*, 2019; Zhao *et al.*, 2020; Pagar *et al.*, 2021).

Site-selective incorporation of ncAAs into recombinant proteins has undergone significant development in recent years (Chin, 2017). The most common method involves using engineered aminoacyl-tRNA synthetases (aaRSs) to selectively charge orthogonal tRNAs with the desired ncAA, which can then be incorporated by the ribosome into proteins in response to a reassigned codon. Orthogonality of these engineered components to the translation machinery of the expression host is essential to ensure reliable ncAA incorporation only at the desired position. This is achieved by employing iterative rounds of positive and negative selections that link cell survival to the activity and selectivity of the engineered aaRS (Liu and Schultz, 2010). Hundreds of aaRS-tRNA systems that incorporate diverse ncAAs have been generated in this way, demonstrating the success and broad applicability of this approach (Huang and Liu, 2018). The maturation of ncAA incorporation technology has led to the development of powerful methods for investigating and modulating protein structure and function. For example, the synthesis, localisation and functional properties of proteins have been interrogated using biorthogonal handles (Evans *et al.*, 2019), spectroscopic probes (Chatterjee *et al.*, 2013) and mimics of post-translational modifications (Chen *et al.*, 2018). Genetic code expansion technology can also be applied in enzymology and enzyme engineering. For example, ncAA incorporation provides new opportunities to decipher the sophisticated catalytic mechanisms of enzymes (Onderko *et al.*, 2017; Ortmayer *et al.*, 2020, 2021). An exciting and ambitious application of genetic code expansion is to create new catalytic mechanisms within protein active sites, leading to new enzyme functions that would be challenging to create using only canonical amino acids side chains (Drienovská *et al.*, 2018; Liu *et al.*, 2018; Burke *et al.*, 2019). Here, we review selected examples from the past five years to illustrate progress in the field.

## Probing and augmenting enzyme mechanisms

**Fig. 1:**
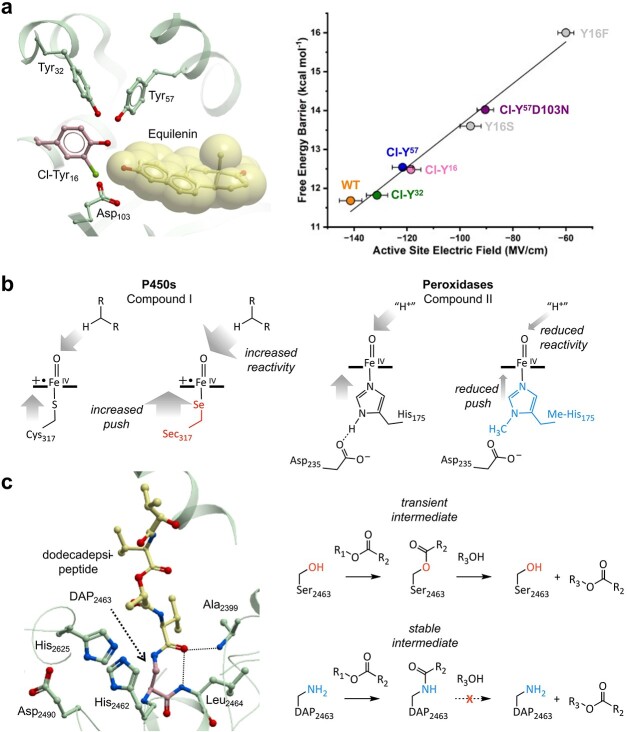
Probing and augmenting enzyme mechanisms using ncAAs. (**a**) Left: crystal structure of ketosteroid isomerase containing a 3-chlorotyrosine residue (Cl-Tyr16, pink carbons) in complex with the substrate equilenin (shown as atom coloured ball and sticks and Corey–Pauling–Koltun spheres with yellow carbons, pdb code: 5KP1). Key residues are shown as atom coloured ball and sticks with green carbons. Right: plot of enzymatic free energy barrier versus active site electric field strength experienced by the equilenin C=O group for selected ketosteroid isomerase variants (Wu and Boxer, 2016). (**b**) Schemes showing Compounds I and II reactivities in P450s and peroxidases, respectively, illustrating the power of ncAAs to tune proximal ligand electron donation (the ‘push’) and control ferryl reactivity (Onderko *et al.*, 2017; Ortmayer *et al.*, 2020, 2021). (**c**) Left: crystal structure of Vlm TE domain with bound dodecadepsipeptide shown as atom coloured ball and sticks with yellow carbons (pdb code: 6ECF). Key residues are shown as atom coloured ball and sticks with green carbons, including non-natural DAP2463 (pink carbons). Right: the active site Ser2463 of the Vlm TE domain rapidly conjugates depsipeptidyl substrates (R_1_OCOR_2_, R_3_OH) via a transient acyl-enzyme intermediate. Substitution of Ser2463 with DAP leads to formation of stable amide intermediates that are resistant to hydrolysis (Huguenin-Dezot *et al.*, 2019).

Site-directed mutagenesis has been used by biochemists for several decades as a means of interpreting the roles of key catalytic residues in enzymes. However, mutagenesis is restricted to the limited number of available amino acids defined by nature’s genetic code. Standard mutation of key catalytic residues often renders an enzyme inactive, limiting the degree of information that can be gained through this approach. Moreover, substitutions designed to probe important biological interactions (e.g. hydrogen bonds or π–π interactions) can often lead to significant structural perturbations, complicating the interpretation of structure–function relationships. The availability of an expanded genetic code provides a more precise means of probing biological mechanisms by allowing more subtle substitutions of individual atoms or functional groups within the active sites of proteins of interest. Early successful examples of this approach include the use of fluorinated tyrosines to probe proton-coupled electron transfer processes in ribonucleotide reductases (Seyedsayamdost *et al.*, 2006). Tyrosine analogues have also been used to interrogate cation–π interactions in squalene cyclases and post-translational modifications in metalloenzymes (Morikubo *et al.*, 2006; Zhou *et al.*, 2013; Li *et al.*, 2019; Yu *et al.*, 2022). Incorporation of chlorinated tyrosines in ketosteroid isomerase has been used to modulate the electric field applied to the substrate carbonyl without perturbing the delicate hydrogen bond network in the active site (Fig. 1a; Wu and Boxer, 2016). This approach enabled the identification of a linear correlation between electric field strength and the activation energy barrier for substrate isomerization, confirming the importance of electrostatic stabilisation in the catalytic mechanism.

Non-canonical substitutions have also proven to be valuable tools for examining the role of axial ligands in heme enzymes. For example, replacement of axial thiolates with selenocysteine residues has been used to probe the catalytic mechanism of P450 monooxygenases (Sivaramakrishnan *et al.*, 2012; Onderko *et al.*, 2017). By comparing the C-H abstraction kinetics of cysteine and selenocysteine ligated CYP119, the proposed link between stronger axial ligand electron donation and increased Compound I reactivity could be experimentally validated (Fig. 1b; Onderko *et al.*, 2017). More recently, an expanded amino acid alphabet has been exploited to introduce functional analogues of histidine as axial ligands in heme peroxidases, revealing new insights into the role played by the ‘imidazolate-like’ proximal histidine ligand in controlling ferryl reactivity (Green *et al.*, 2016; Ortmayer *et al.*, 2020). An engineered pyrrolysyl-tRNA synthetase/pyrrolysyl-tRNA pair was used to replace the axial His ligand of cytochrome *c* peroxidase (C*c*P) by N_δ_-methylhistidine (Me-His) (Xiao *et al.*, 2014; Ortmayer *et al.*, 2020). Histidine methylation disrupts the hydrogen bond between the proximal ligand and the neighbouring aspartate, an interaction which is known to increase the electron-donating properties of the histidine ligand (De Visser *et al.*, 2003). Comparison of C*c*P His and C*c*P Me-His crystal structures revealed this axial ligand substitution to be a structurally conservative modification, with the active site geometry and conformations of key residues well preserved. In contrast to the findings in P450s, ligand substitution in C*c*P had negligible effects on Compound I reactivity. Instead, the rate-limiting Compound II reduction step is 10-fold slower in C*c*P Me-His, with weaker electron donation from the axial Me-His (‘the push’) creating an electron-deficient ferryl oxygen with reduced proton affinity (‘the pull’) (Fig. 1b; Ortmayer *et al.*, 2020).

Further evidence of the link between ferryl p*K*a and Compound II reactivity was provided by substituting Trp51 of C*c*P with the tryptophan analogue 3-benzothienyl-L-alanine (S-Trp) (Englert *et al.*, 2015; Ortmayer *et al.*, 2021). The N-H group of Trp51 forms a hydrogen bond to the ferryl oxygen (Meharenna *et al.*, 2010), an interaction which is also present in ascorbate peroxidase but absent in many heme peroxidases, including the prototypical peroxidase from horseradish (Berglund *et al.*, 2002; Sharp *et al.*, 2003). The Trp51S-Trp substitution provides a structurally conservative means of removing this hydrogen bonding interaction, leading to a more basic and reactive Compound II, which manifests in a >60-fold increase in the catalytic activity towards non-biological oxidations of small phenolic substrates (Ortmayer *et al.*, 2021).

Non-canonical ligand substitutions have also been used to enhance the promiscuous activities of non-catalytic heme proteins. Replacement of the axial His ligand of the oxygen-binding protein myoglobin (Mb) with a less electron-donating Me-His led to a five-fold increase in *k*_cat_ for guaiacol oxidation (Pott *et al.*, 2018). This increased activity correlates with an increase in the second-order rate constant for the formation of the ferryl intermediate, likely resulting from a more electrophilic iron centre in the Me-His ligated protein. The activity of Mb Me-His was further enhanced through the targeted introduction of distal pocket mutations that had previously been shown to improve Mb peroxidase catalysis, coupled with iterative rounds of directed evolution using workflows adapted to an expanded genetic code (Ozaki *et al.*, 1997). This engineering afforded a highly active MbQ2.2 Me-His variant with a catalytic efficiency for guaiacol oxidation that is 1140-fold higher than wild-type Mb and only two-fold lower than horseradish peroxidase (Pott *et al.*, 2018). Me-His ligand substitutions in Mb variants have also led to the generation of artificial carbene transferases with improved catalytic properties (Hayashi *et al.*, 2018; Carminati and Fasan, 2019; Chandgude *et al.* 2019). Ligand substitution in a His46Val Val68Ala Mb variant (Mb^*^) gave modest improvements in conversions in cyclopropanation reactions of styrene with ethyl diazoacetate in the presence of reductant under established anaerobic conditions (Hayashi *et al.*, 2018). More remarkably, ligand substitution in Mb^*^ alleviates the need for exogeneous dithionite reductant and allows for efficient catalysis under aerobic conditions. These augmented properties were attributed to the increased electrophilicity of the Fe(III) centre when coordinated by the Me-His ligand (Hayashi *et al.*, 2018). To further expand the catalytic repertoire of Mb based carbene transferases, the Fasan group combined a non-canonical Me-His ligand with a non-native electron-deficient heme analogue in Mb^*^ (Carminati and Fasan, 2019). The resulting artificial carbene transferase can efficiently promote cyclopropanations of electron-deficient alkene substrates, which have proven to be challenging with previously engineered carbene transferases. This expanded reaction scope was attributed to a radical-type carbene transfer mechanism stemming from the combined effect of the electron-deficient heme and non-natural axial ligand.

Many hydrolytic and acyl transfer enzymes, such as esterases and amidases, operate via acyl-enzyme intermediates (Otto and Schirmeister, 1997; Long and Cravatt, 2011; Swatek and Komander, 2016). These intermediates are only formed transiently during catalysis, which makes them difficult to isolate and study (Yang and Drueckhammer, 2001). Previously, substrate analogues and active site mutations have been used to stabilise these intermediates, however, these complexes are not necessarily representative of those generated in natural systems (Liu *et al.*, 2006; Cappadocia and Lima, 2018). The Chin group showed that replacing the nucleophilic serine or cysteine residues with an isosteric 2,3-diaminopropionic acid (DAP) leads to the formation of a stable amide bond between the enzyme and substrate, enabling the trapping and characterisation of near-native acyl-enzyme species (Huguenin-Dezot *et al.*, 2019). A pyrrolysyl-tRNA synthetase was evolved to accept a photocaged version of DAP which, upon successful incorporation into a recombinant protein, could be deprotected with light irradiation to yield the free primary amine (Huguenin-Dezot *et al.*, 2019). This incorporation strategy was then used to interrogate the catalytic cycle of valinomycin synthetase (Vlm), a member of the nonribosomal peptide synthetase family. The thioesterase (TE) domain of Vlm is responsible for oligomerising and cyclising linear depsipeptidyl substrates to produce the final product valinomycin (Magarvey *et al.*, 2006; Jaitzig *et al.*, 2014). Replacement of the active site serine of the TE domain with DAP and subsequent incubation with model substrates generated stable acyl-enzyme intermediates that could be isolated and structurally characterised (Fig. 1c), revealing important conformational changes in the lid element of Vlm TE when full-length depsipeptides were conjugated to the enzyme (Huguenin-Dezot *et al.*, 2019). These conformational changes are likely responsible for pre-organisation of the substrate into a productive pose for cyclisation. The DAP incorporation strategy was subsequently employed to characterise hydrolase and protease substrate specificities by mechanistic trapping of intermediates *in vivo* (Tang *et al.*, 2022).

## Embedding new catalytic mechanisms into proteins

Recent advances in computational design have enabled the development of *de novo* enzymes with non-biological mechanisms (Huang *et al.*, 2016; Lovelock *et al.*, 2022). Enzyme design coupled with directed evolution has afforded proficient biocatalysts for a number of transformations (Röthlisberger *et al.*, 2008; Althoff *et al.*, 2012; Crawshaw *et al.*, 2021); however, one enzyme class that has proven to be challenging to design are hydrolases (Richter *et al.*, 2012; Rajagopalan *et al.*, 2014). Many natural hydrolytic enzymes feature active sites containing a catalytic nucleophile that is activated by an adjacent basic residue (Brady *et al.*, 1990; Smith *et al.*, 2008). Efforts to recapitulate this mechanistic motif computationally commonly result in proteins where catalysis stalls due to the formation of stable acyl-enzyme complexes that are resistant to hydrolysis (Richter *et al.*, 2012; Rajagopalan *et al.*, 2014).

**Fig. 2:**
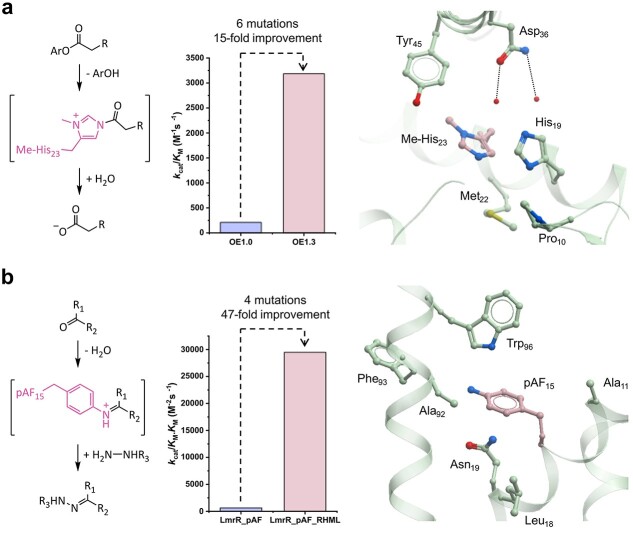
Non-biological mechanisms in *de novo* enzymes. (**a**) Left: OE1.0 promotes ester hydrolysis via the formation of transient acyl-imidazolium intermediates. Middle: bar chart comparing the *k*_cat_/*K*_M_ for fluorescein 2-phenylacetate hydrolysis by OE1.0 and the evolved variant OE1.3. Right: crystal structure of OE1.3 (pdb code: 6Q7Q), highlighting key active site residues (shown as atom coloured ball and sticks with green carbons) including the Me-His nucleophile (pink carbons). Hydrogen bonding interactions between Asn46 and two ordered water molecules are also shown (Burke *et al.*, 2019). (**b**) Left: LmrR_pAF uses a reactive pAF residue to promote hydrazone and oxime formations. Middle: bar chart comparing the *k*_cat_/*K*_M_.*K*_M_ for 4-hydrazino-7-nitro-2,1,3-benzoxadiazole condensation with 4-hydroxybenzaldehyde by LmrR_pAF and the evolved variant LmrR_pAF_RHML. Right: crystal structure of LmrR_pAF (pdb code: 6I8N), highlighting selected active site residues (shown as atom coloured ball and sticks with green carbons) and the pAF catalytic nucleophile (pink carbons) (Mayer *et al.*, 2019).

To address these limitations, our group exploited an expanded genetic code to reengineer the computationally designed protein BH32 into an efficient hydrolase (Burke *et al.*, 2019). BH32 was originally designed to catalyse the Morita-Baylis-Hillman reaction using a histidine nucleophile at position 23 (Bjelic *et al.*, 2013). Using activated esters as substrates, BH32 was also found to display a promiscuous hydrolase activity, exhibiting the classical biphasic reaction profile reminiscent of previously designed hydrolases (Burke *et al.*, 2019). To unlock the catalytic activity, the His23 nucleophile was replaced by Me-His using an engineered aaRS/tRNA pair (Xiao *et al.*, 2014). Upon acylation of the Me-His nucleophile, a charged and highly reactive acyl imidazolium intermediate is generated, which facilitates downstream hydrolysis (Fig. 2a; Burke *et al.*, 2019). This mechanistic strategy is similar to that employed in small-molecule organocatalysis using catalytic nucleophiles such as 4-dimethylaminopyridine (Wurz, 2007). As intended, replacement of His by Me-His resulted in an enzyme (OE1.0) with improved hydrolytic turnover. This activity was further enhanced over iterative rounds of directed evolution using a spectrophotometric assay. After three rounds, an OE1.3 variant emerged with six mutations clustered around the active site. Kinetic studies revealed that the catalytic efficiency of OE1.3 is 15-fold higher than the parent template OE1.0 and 2800-fold higher than the organocatalyst 4-dimethylaminopyridine (Burke *et al.*, 2019). Crystal structures of OE1.0 and OE1.3 shed light on the active site features that emerged to support catalysis by the Me-His nucleophile. Interestingly, an inhibited structure of OE1.3 suggests that the Leu10Pro mutation installed during the final round of evolution conferred functionally relevant domain motions to enhance hydrolase activity (Griffin *et al.*, 2012). While OE1.3 was found to possess increased activity towards a range of substrates featuring phenyl, furanyl and napthylic moieties, its activity and selectivity towards chiral substrates containing α-methyl substituents were low (Burke *et al.*, 2019). To address this limitation, further evolution was performed to generate variant OE1.4, which displayed substantially higher activity and enantioselectivity towards these chiral substrates, demonstrating that designed enzymes with an expanded set of amino acids can be engineered to perform enantioselective transformations.

The Roelfes lab have employed *p*-aminophenylalanine (pAF) as a non-canonical catalytic nucleophile to promote a range of transformations in designer enzymes (Drienovská *et al.*, 2018). Hydrazone and oxime formations were selected as initial target transformations based on precedent for accelerating these reactions using aniline as an organocatalyst (Dirksen *et al.*, 2006a, b). The multidrug transcriptional regulator from *Lactococcus lactis* (LmrR), a small homodimeric protein with a large hydrophobic pore, was selected as a protein scaffold (Madoori *et al.*, 2009). The substrate binding promiscuity of LmrR makes this an attractive scaffold for generating enzymes with new catalytic activities (Bos *et al.*, 2015; Drienovská *et al.*, 2015). A pAF residue was introduced at position 15 using a two-stage protocol involving the genetic encoding of a *p*-azidophenylalanine followed by chemical reduction with tris(2-carboxyethyl)phosphine to unmask the reactive aniline side chain. X-ray crystallography confirmed that the pAF side chain was positioned close to Trp96, which contributes to the recognition of aromatic substrates in the LmrR binding pocket (Fig. 2b). Although the initial LmrR_pAF15 variant gave only modest activity improvements over the parent protein LmrR, subsequent optimization through targeted rounds of directed evolution afforded a quadruple mutant with a 55-fold improvement in *k*_cat_ and a 26,000-fold increased efficiency over aniline in solution (Fig. 2b). Introduction of A11L and N19M mutations are thought to play a role in positioning the aniline side chain in a productive pose for catalysis. The reaction scope of LmrR_pAF variants has subsequently been extended to include enantioselective Friedel-Crafts alkylations of indoles and enantioselective Michael additions of enals and 2-acylimidazoles (Zhou and Roelfes, 2020; Leveson-Gower *et al.*, 2021, 2022). In the latter case, catalysis is dependent upon the synergistic action of the pAF nucleophile and a Cu(II)-phenanthroline complex that is sandwiched between two tryptophan residues.

## Conclusions

Integrating ncAAs into enzyme engineering and design methodologies unlocks a wealth of new opportunities in biocatalysis research. The studies outlined in this review highlight how the availability of an expanded alphabet of amino acids can provide new avenues to explore enzyme mechanisms, enhance biocatalyst properties and design enzymes with new catalytic functions which would be challenging to access using the standard set of canonical amino acids. Importantly, the properties of enzymes made using an expanded genetic code can be tuned and optimised through directed evolution to deliver proficient and selective biocatalysts. Moving forward, continued expansion of the genetic code will allow the installation of a greater diversity of ncAAs with interesting functional side chains. Access to these new functional elements will enable the development of enzymes with entirely new modes of catalysis. For example, recent studies have shown how the introduction of photosensitizers can open up new photochemical processes mediated by proteins (Liu *et al.*, 2018; Sun *et al.*, 2022; Trimble *et al.*, 2022). Although more technically challenging, strategies for selectively embedding two or more ncAAs will further expand the repertoire of catalytic mechanisms conceivable within protein active sites. To translate fundamental findings into larger-scale biocatalytic applications, it will be important to develop efficient and cost-effective methods of producing enzymes containing ncAAs on scale. To this end, engineering more efficient orthogonal translation components and employing engineered or synthetic strains specifically tailored to maximise ncAA incorporation efficiency will enable modified protein production in high titres using low concentrations of ncAAs. Engineering strains that produce the target ncAA biosynthetically will lead to further cost reductions. For these reasons, we are optimistic that genetic code expansion methodologies will play an increasingly prominent role in fundamental and translational biocatalysis research in the coming years.

## Notes

**Edited by:** Christopher Snow
